# Traumatic dental injuries in a university hospital: a four-year retrospective study

**DOI:** 10.1186/s12903-015-0124-5

**Published:** 2015-11-04

**Authors:** Benjamin Mahmoodi, Roman Rahimi-Nedjat, Jens Weusmann, Adriano Azaripour, Christian Walter, Brita Willershausen

**Affiliations:** Department of Operative Dentistry and Periodontology, University Medical Center of the Johannes Gutenberg-University Mainz, Augustusplatz 2, 55131 Mainz, Germany; Department of Oral and Maxillofacial and Facial Plastic Surgery, University Medical Center of the Johannes Gutenberg-University Mainz, Augustusplatz 2, 55131 Mainz, Germany

**Keywords:** Dental trauma, Dentoalveolar-trauma, Prevalence, Tooth injury

## Abstract

**Background:**

Traumatic dental injuries present complex injuries of the dentoalveolar system. Aim of this study was to investigate the frequency and patterns of traumatic dental injuries in a University dental emergency service over four years.

**Methods:**

A retrospective investigation on all dental trauma patients presenting at the dental emergency service of the University Medical Center Mainz, Germany between 01/2010 and 12/2013 was conducted. Demographic data, the cause and type of trauma and the initial therapy were analyzed.

**Results:**

Out of 16,301 patients, 1,305 patients (8 %; average age 14.7 years ±15.7; 60.1 % male, 39.9 % female) came due to trauma. 63.9 % of the traumas occurred on weekends. The most frequent reason for injuries was falls (54.6 %). No correlation could be found between the cause and the kind of trauma. In 48.6 % of the cases only one tooth was involved, in 33.5 % two. The permanent dentition was traumatized in 56.6 % of cases, the deciduous teeth in 41.1 %. The most frequently affected tooth was the central upper incisor (61.0 %). Hard-tissue injuries were significantly more frequent in the permanent dentition, while periodontal injuries were seen significantly more often in the deciduous dentition.

**Conclusion:**

Eight percent of all patients seeking help at the dental emergency service presented with trauma, meaning that dental traumatology is one of the major topics in emergencies. To improve the quality of care, further public education, expert knowledge among dental professionals and a well-structured emergency service are necessary.

## Background

Traumatic dental injuries (TDIs) often present as serious and complex injuries of the dentoalveolar system. Prevalences vary depending on cultural and social factors [1–3]. Mostly, TDIs occur at a young age, but they are observed in any age group [3, 4]. Factors associated with higher prevalences of dental injuries are increased overjet, class II type malocclusion, having orthodontic needs, and male gender [5, 6].

Studies show that dental trauma accounts for about 5 % of all injuries leading to inpatient or outpatient treatment and that the oral region is the sixth most frequently injured part of the body [7, 8]. Mostly, TDIs involve anterior teeth and represent painful events that may result in complications such as crown discoloration, pulp necrosis, apical periodontitis, ankyloses, and inflammatory root resorption and tooth loss as a consequence of the above mentioned complications or primary event [9]. In addition to functional problems, traumatic dental injuries (TDIs) may cause aesthetic, psychological and social problems by affecting the appearance and speech of patients [10]. To minimize complications and to save the affected tooth, immediate and appropriate management is required.

The incidence of TDIs is higher in the late evenings and on weekends, which is associated with the lifestyle [7, 11]. Consequently many patients present in dental emergency service units since they usually operate outside the dentists’ regular clinical hours. Available data on the frequency and patterns of dental traumas in Germany are sparse but necessary to provide recommendations for prevention and improvement in the quality of the therapy.

As our institution is one of two university medical centers in the Rhine-Main-metropolitan region, which has 5.5 million inhabitants, our dental clinic is frequently sought out by dental emergency patients and in fact sees these patients daily. The aim of this retrospective study was to investigate the frequency of TDIs among all patients who presented to the dental emergency service within a period of four years.

## Methods

All patients that presented in the dental emergency outpatient department of the University Medical Center of the Johannes Gutenberg-University in Mainz between January 2010 and December 2013 were included. Altogether, 16,301 patients were seen in the dental emergency outpatient department in the four-year period (average age 35.3 years ±19.5, 54.4 % male [34.9 years ± 19.1] and 45.6 % female [35.8 years ± 19.9]). A retrospective investigation on these patients was then carried out concerning epidemiologic factors, the cause and the type of trauma as well as concomitant soft tissue injuries and the initial therapy. For this, electronic health records were reviewed and subjected to further analysis. Inpatient cases that had already had treatment in other departments, such as the departments for oral and maxillofacial surgery, trauma surgery, and neurosurgery, due to more severe injuries were excluded since these patients are usually transferred or presented at a later stage to the dental department and could thereby introduce a bias regarding the prognosis and sufficient treatment of the affected teeth.

Due to the hospital laws of each state in Germany (Landeskrankenhausgesetz), no ethical approval is necessary in retrospectively performed studies evaluating patient data that already exist. All patients were informed of the anonymized use of their records at the time they contacted the hospital for dental care.

### Classification of dental injuries

Traumatic dental injuries were classified as follows [12]: (i) enamel crack, (ii) enamel fracture, (iii) enamel-dentin fracture without pulp exposition, (iv) enamel-dentin fracture with pulp exposition, (v) vertical crown-root fracture, (vi) root fracture, (vii) concussion, (viii) subluxation, (ix) lateral luxation, (x) intrusion and (xi) avulsion. (i)-(iv) were summarized as hard tissue injuries, (v) and (vi) as root fractures and (vii)-(xi) as periodontal injuries.

### Statistical analysis

For statistical analysis, Microsoft Excel 2010 (Redmond, WA, USA) and SPSS 22 (IBM, Armonk, NY, USA) were used. The chi-square test was used for analysis of uneven distributions between two groups, such as the gender distribution and the distributions of the different kinds of traumas in relation to the dentition and/or the cause of trauma.

## Results

**Fig. 1:**
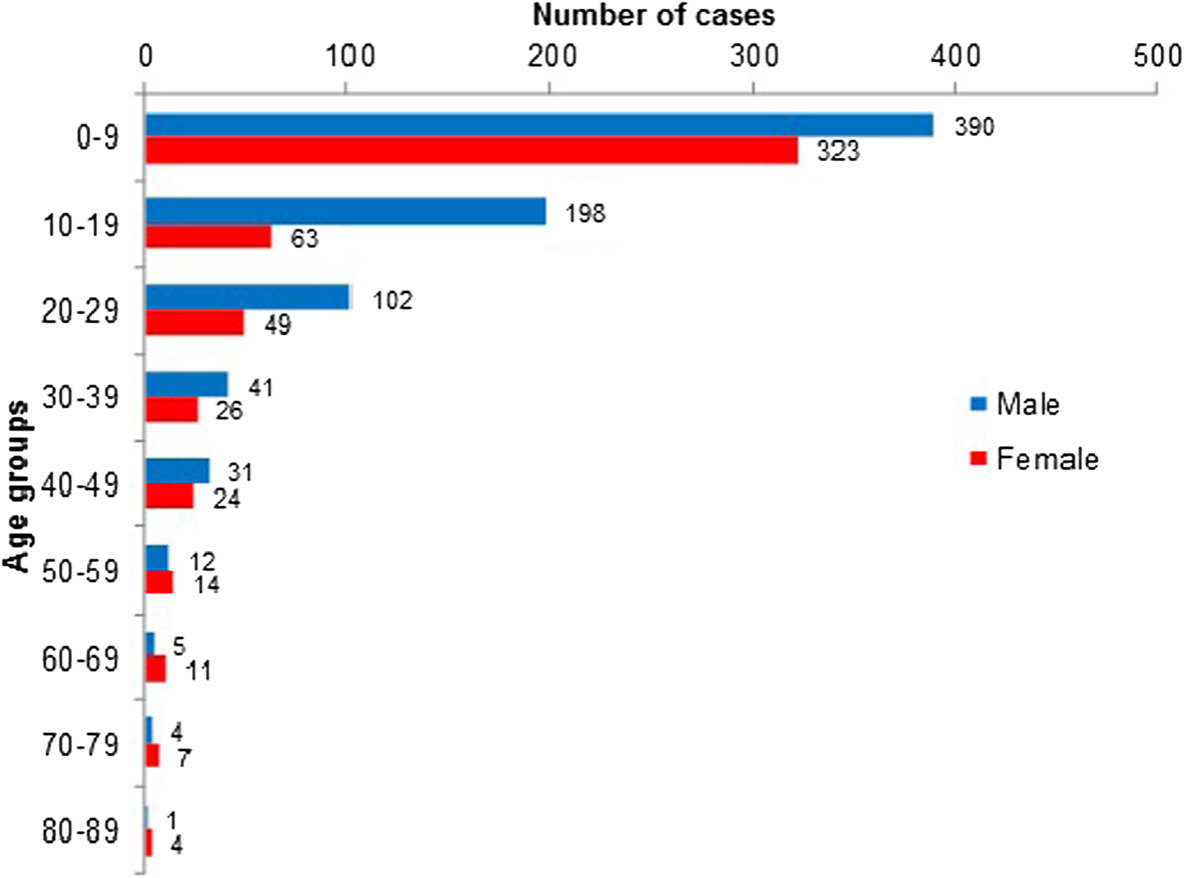
Age and gender distribution of trauma patients

Out of the 16,301 patients seen in the four-year period, 8.0 % presented with TDIs (*n =* 1305; average age 14.7 years ±15.7, 60.1 % male [14.4 years ± 13.8] and 39.9 % female [15.2 years ± 18.2]), with a male to female ratio of 1.5:1. Men were significantly more often affected than females (*p <* 0.001). The youngest patient presented at an age of 7 months, while the oldest patient was 88 years. More than half of the trauma collective (54.6 %; *n =* 713) was under the age of ten (Fig. 1).

**Fig. 2:**
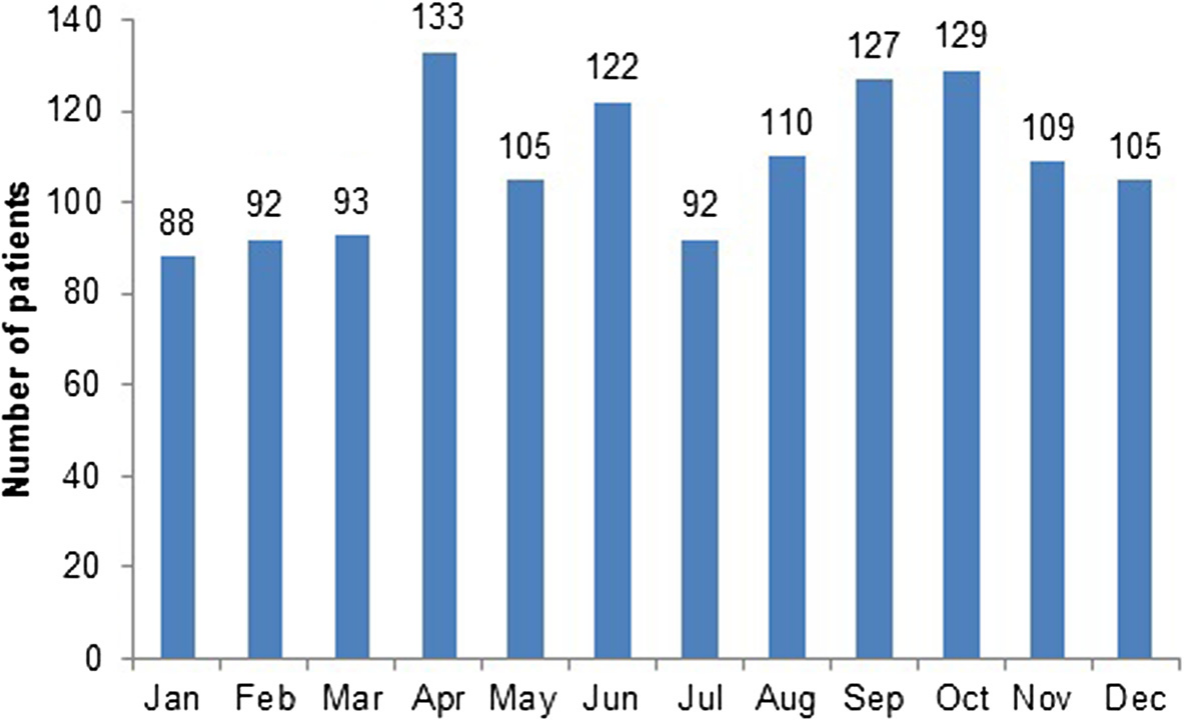
Distribution of TDIs among the months of the year

**Fig. 3:**
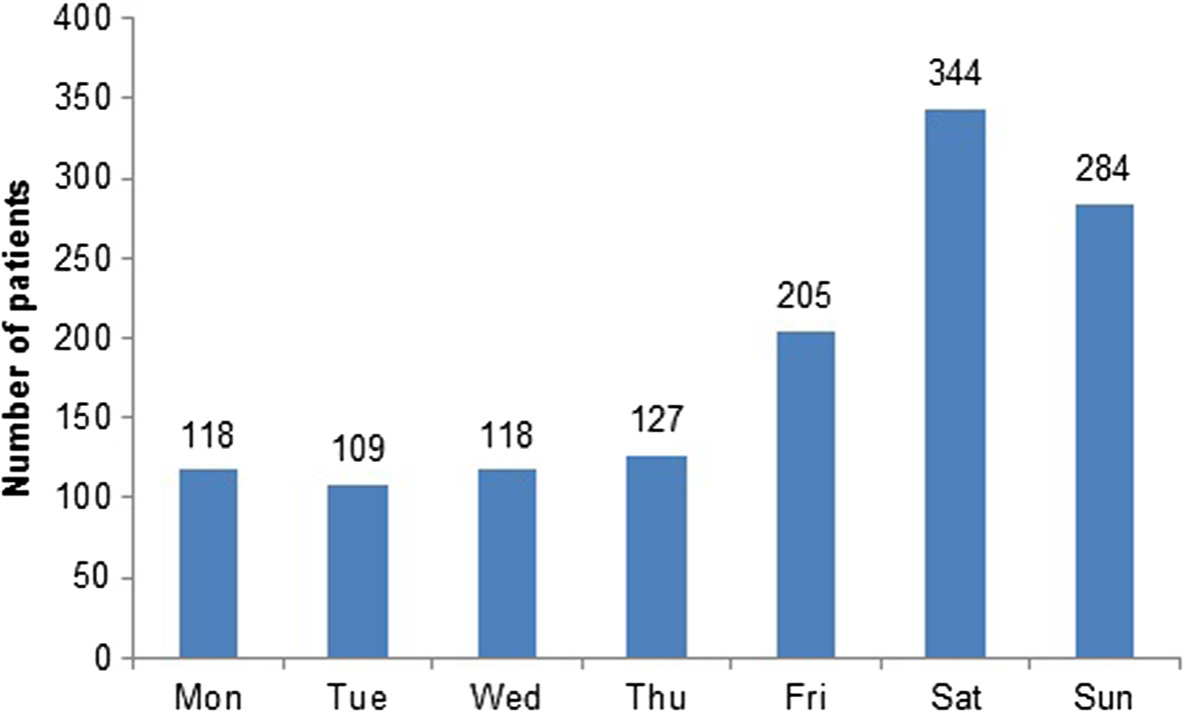
Distribution of TDIs among the days of the week

Concerning the year and month of visit, a homogeneous distribution of trauma cases was found (Fig. 2). In 2010, 280 patients were treated, in 2011 354, in 2012 349 and in 2013 322 patients. Nearly two-third (63.9 %; *n =* 833) of all TDIs occurred on weekends (Fig. 3). 74 patients (5.7 %) came on a public holiday. The most frequent reasons for TDIs (Table 1) were falls (54.6 %; *n =* 713), followed by sport accidents (13.4 %), and recreational accidents (8.8 %). Most of the sport accidents occurred in the patient group aged between 10 and 19 years (46.3 %), followed by the 0–10 year-olds (30.3 %). Significant differences were seen in the frequency of the causes between the genders. While assaults were more often the trauma reason in male patients (*p <* 0.001), the frequency of falls was higher among females (*p <* 0.001). Most assaults occurred in the 20–29 year age group (48.3 %), followed by the 10–19 year-olds (31 %).

**Table 1:** Causes of trauma

Cause of trauma	Number of patients *n* (%)	Male *n* (%)	Female *n* (%)	Permanent dentition *n* (%)	Decidious dentition *n* (%)
*Total*	*1305 (100)*	*784 (100)*	*521 (100)*	*739 (100)*	*537 (100)*
Falls (domestic)	257 (19.7)	144 (18.4)	113 (21.7)	76 (10.3)	172 (32.0)
Falls (outdoors)	456 (34.9)	228 (29.1)	228 (43.8)	214 (29.0)	236 (43.9)
Work accident	22 (1.7)	17 (2.2)	5 (1.0)	22 (3.0)	0
Sport accident	175 (13.4)	128 (16.3)	47 (9.0)	146 (19.8)	25 (4.7)
Assaults	87 (6.7)	79 (10.1)	8 (1.5)	81 (11.0)	5 (0.9)
Traffic accident	81 (6.2)	56 (7.1)	25 (4.8)	66 (8.9)	12 (2.2)
Recreation accident	115 (8.8)	66 (8.4)	49 (9.4)	65 (8.8)	47 (8.8)
Other	112 (8.6)	66 (8.4)	46 (8.8)	68 (9.2)	40 (7.4)

Among the 1,305 patients, 2,319 teeth were traumatized (mean: 1.8 teeth per patient). In 48.6 % (*n =* 634) of the cases, only one tooth was involved; in 33.5 % (*n =* 437) two teeth were injured, and in 16.7 % (*n =* 216) three or more teeth were injured. 337 patients (25.8 %) had a concomitant soft tissue injury. 18 patients (1.4 %) presented with a soft-tissue injury without dental involvement.

**Fig. 4:**
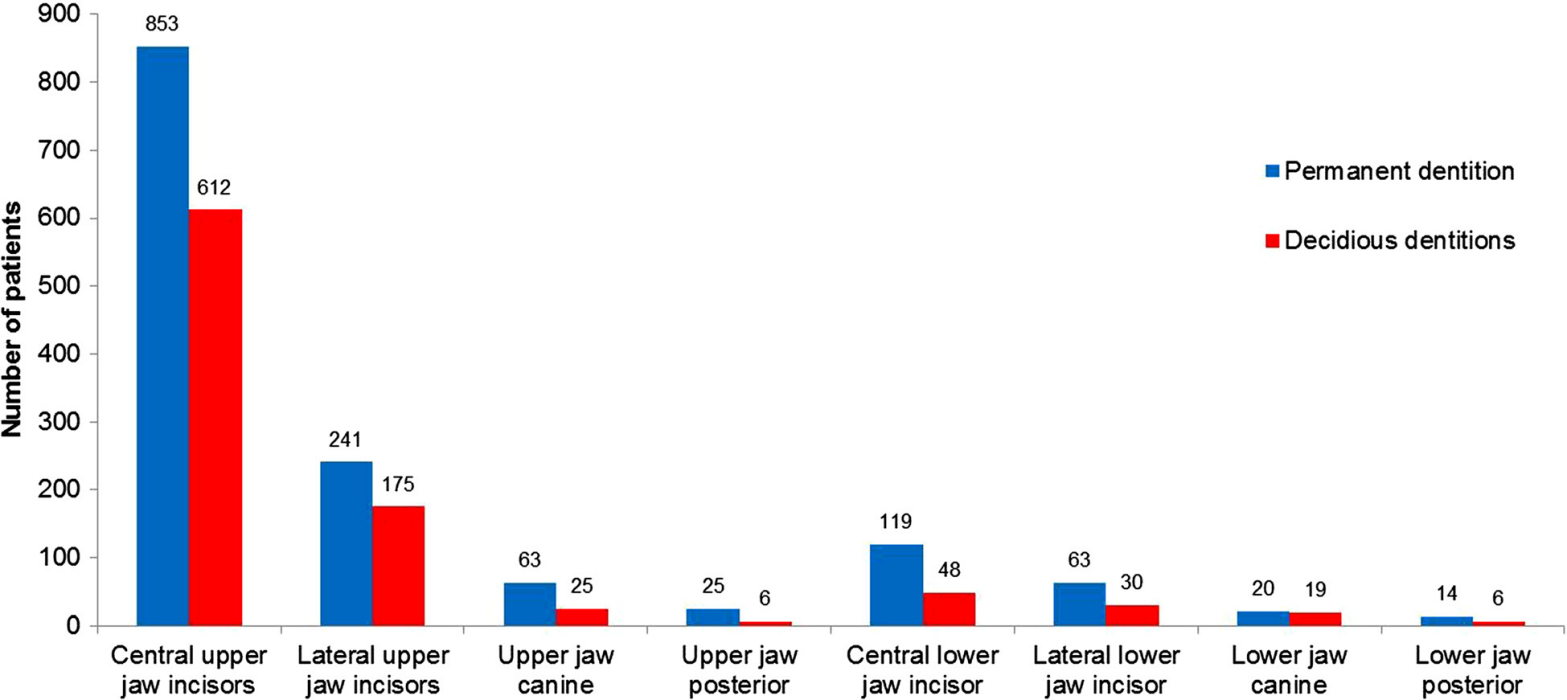
Affected dentition

The permanent dentition was traumatized in 56.6 % (*n =* 739) of cases, the deciduous teeth were traumatized in 41.1 % (*n =* 537), and eleven patients (0.8 %) had trauma to the transitional dentition with affection of the deciduous and permanent dentition (Fig. 4).

### Permanent dentition

**Fig. 5:**
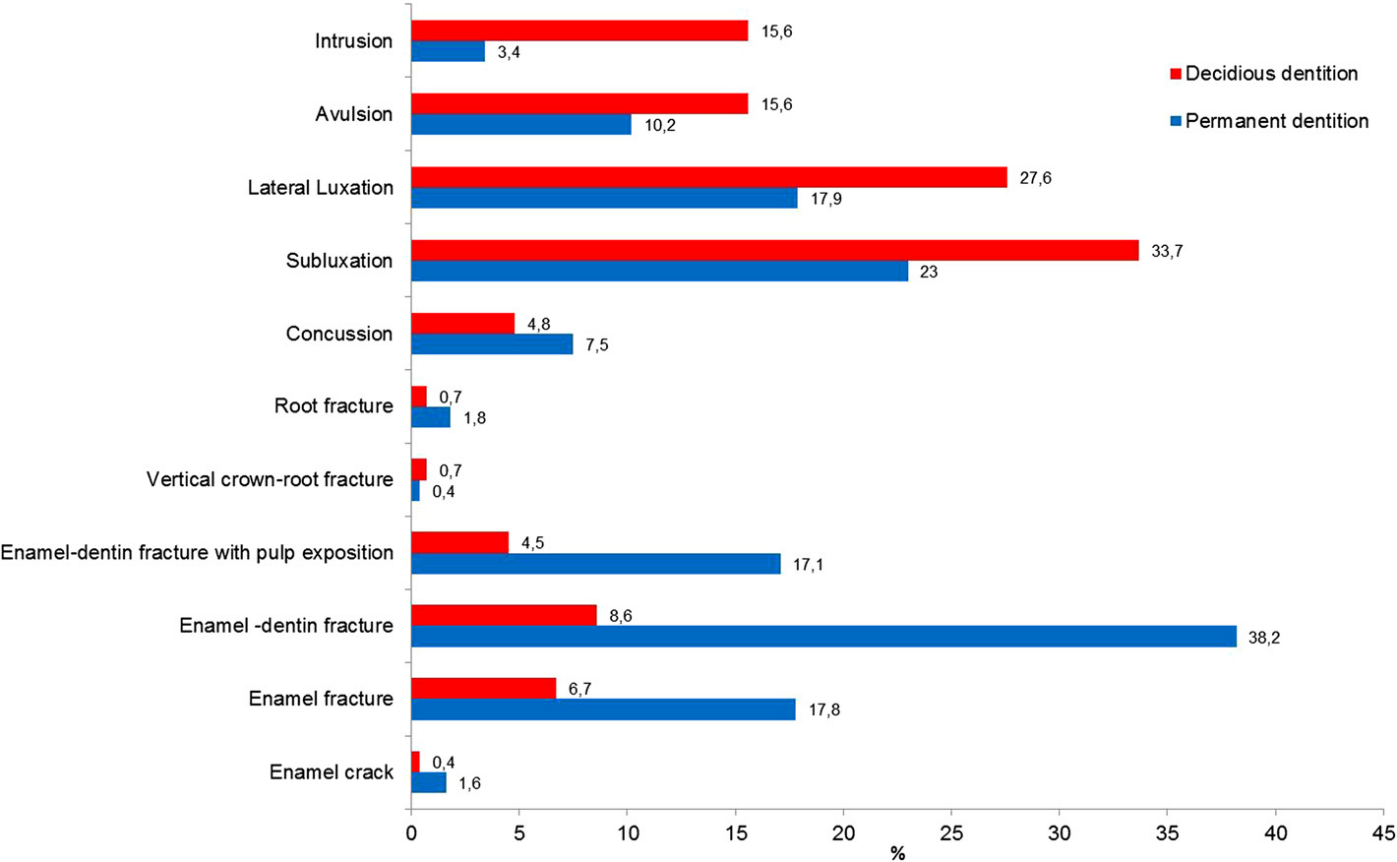
Distribution of type of trauma

Among the 739 patients with permanent dentition, the average age was 23.0 years ±16.5 (63.1 % male [21.2 years ± 13.9] and 36.9 % female [26.0 years ± 19.7]), and the male to female ratio was 1.7:1. With a total of 1398 injured teeth, there was an average of 1.9 permanent dentition injuries per patient. 84.5 % of the traumatized teeth were located in the upper jaw. The most frequently affected tooth was the central upper incisor (61.0 % *n =* 853, Fig. 4). Enamel dentin-fracture was the most frequently diagnosed condition (38.2 %), followed by subluxation (23.0 %) and lateral luxation (17.9 %, Fig. 5).

### Decidious dentition

Among the 537 patients with deciduous dentition, the average age was 3.6 years ±2.0 (55.7 % male [3.7 years ± 2.3] and 44.3 % female [3.4 years ± 1.7]), and the male to female ratio was 1.3:1. The mean number of injured teeth per patient was 1.7 (921 teeth in 537 patients). 88.2 % of traumatized teeth were located in the upper jaw. Subluxation (33.7 %) was the most common diagnosis, followed by lateral luxation (27.6 %), avulsion (15.6 %) and intrusion (15.6 %, Fig. 5).

### Type of trauma

No correlation could be found between the cause of the trauma and the type of TDIs, but a comparison of the different dentitions showed significant differences in the type of trauma. While hard-tissue injuries appeared significantly more often in patients with permanent dentition (*p <* 0.001), periodontal injuries were found to be more frequent in the deciduous dentition (*p <* 0.001). There was no statistical difference in the distribution of root fractures (*p =* 0.412). 10.2 % (*n =*133) of patients had a combination of hard tissue injury (diagnoses i-iv) and periodontal injury (diagnoses vii-xi), while the most common combination of diagnoses was subluxation and uncomplicated enamel-dentin-fracture (4.1 %; *n =* 54).

### Initial therapy

In 60.2 % of cases a dental, surgical and/or medical treatment was provided. The treatment measures are summarized in Table 2. As a matter of course, the remainder of the patients was seen by the dentist on duty, but no interventional therapy was indicated or possible.

**Table 2:** Provided treatment

	Soft tissue injury	Hard tissue injury	Root fractures
Temporary filling	52	287	4
Trepanation	12	44	4
Suture	88	70	1
Splinting	220	68	4
Extraction	47	10	6
Presciption of antibiotics	138	59	3
Prescription of analgetics	109	51	2

## Discussion

The prevalence of dental trauma in emergency patients (8 % in the present study) distinctly varies depending on the countries where the studies were conducted. The prevalence is reported to be 66 % in Korea [13], 27.7 % in the United Kingdom [14], 11 % in Greece [15], and 8.4 % in France [16]. This range could be explained by the different health insurance systems; for example, in Germany basic dental treatment and emergency treatment are fully covered by health insurance. Patients in Korea must pay out-of-pocket for emergency treatment, so that only patients with severer problems might visit the clinic [13]. The range of prevalences might also be influenced by different socioeconomic and cultural diversities as well. Although the studies mentioned refer to TDIs in dental trauma, a deeper view on the various kinds, causes and localizations of TDIs was not given.

In contrast to other investigation, no seasonal increase of TDIs during warm months was found [13, 15]. The weekly distribution showed that the major prevalence of TDIs were on Saturdays, followed by Sundays and Fridays. This is associated with intense social activity, sports and leisure time as well as greater alcohol consumption on the weekends. These findings agree with former studies [11, 13, 17].

Men were more likely to visit the dental emergency service due to TDIs than women; men tend to be more prone to trauma than women [4, 9, 11, 18], perhaps because of more violent behavior and participation in more aggressive types of sports [19]. Our results show that the rate of sport accidents as well as assaults was higher among the male collective. The difference in gender was less pronounced in patients with trauma in the deciduous dentition, which is consistent with the literature [20, 21].

An age peak was noticed in the 0–9 year-olds, with a continuous decrease with advancing age. Children are prone to TDIs because of their lack of motor coordination and curious and exploratory behavior [13], and the data presented here are in accordance with the literature [3, 4, 9, 13]. 71–92 % of all TDIs occur before the age of 19 years [3]. The proportion was 74.6 % in this study.

The most common injury in the permanent dentition in our investigation was enamel-dentin-fractures without pulp involvement (38.4 %). This is consistent with the international literature, with a described range between 20.2 % and 51.6 % [4, 9, 18, 22, 23]. In the deciduous dentition, subluxation was the injury most frequently seen (33.7 %). Other studies have shown a similar trend in the distribution of TDIs, with a higher number of soft-tissue injuries in the deciduous dentition and an increase in hard tissue injuries in the permanent dentition [4, 22–24]. Because of the higher elasticity of the supporting tissues, the relatively small roots and reduced alveolar bone support, the deciduous dentition has a predisposition for periodontal injuries, while permanent teeth are embedded more firmly in the alveolar bone and may be more likely to fracture [12]. In addition, the number of luxation injuries might be underestimated since minor luxation in patients with mixed dentition or periodontal disease are difficult to diagnose, and the patients might be less likely to consult a dentist.

Altogether, 1.8 teeth per trauma patient were injured, which is consistent with the range of 1.6 to 1.9 teeth per patient described in the literature [4, 11, 18, 22].

In agreement with the results of previous studies, the central and lateral upper jaw incisors were the teeth most frequently affected [3, 4, 18]. They account for 78.2 % of all injured teeth in the permanent dentition and 85.5 % in the deciduous dentition.

A high share of patients presenting to our emergency dental department did not receive any immediate interventional treatment. Some of these were patients with periodontal injuries in the deciduous dentition that were near exfoliation stage. In such patients, a conservative, “wait-and-watch” philosophy was applied rather than interventional treatment. Some patients presented with minimal hard tissue defects, such as enamel fractures with or without dentine involvement that did not include the pulp. In these patients, no emergency procedures were needed, but they were provided with an appointment at a mutually beneficial time. Certain pediatric patients also did not receive interventional emergency treatment; these were children with isolated dental avulsion injuries who either did not bring the tooth along with them or were cases where the avulsed tooth was a highly resorbed primary tooth that would have exfoliated in due course anyway.

Mostly TDIs happen in everyday life situations, which make them largely unavoidable. As a result, dental trauma is hard to prevent. Educational programs for teachers, parents, caregivers, coaches and paramedics could help to minimize the long term effects of trauma and to achieve better prognoses through immediate and correct treatment. Efforts to reduce environmental (e.g. unsafe school grounds and playgrounds) and behavioral risk-factors (e.g. alcohol consumption, school-bullying, high-risk sport activities, etc.) for TDIs may have an impact on prevention. Despite all efforts, they cannot be avoided in most situations. Sport injuries, which were the reason for trauma in 13.4 % of cases, present opportunities for prevention. The use of mouth guards has been regarded as an effective measure to prevent or reduce the severity of dental trauma in sports [25]. From this perspective, it is important to sharpen about the awareness of the positive effects of these devices among coaches as well as the athletes themselves.

A limitation of the present study is the lack of follow-up. Based on our findings, future studies on clinical outcomes, complications and the long time survival rates could be conducted. However, our data give an overview of the prevalence of TDIs in a German metropolitan region.

The high proportion of weekend injuries that usually need immediate treatment to prevent long-term deficiencies shows the importance of dental emergency units so that patients receive the required care as soon as possible.

## Conclusion

With an 8 % share of all patients presenting to the emergency dental service and the impact on esthetics and function, we think TDIs can be regarded as a legitimate public health issue. To improve the quality of care, further public education, expert knowledge on the diagnosis and treatment of TDIs among dental professionals and a well-structured emergency service will be the key.

## Abbreviations

TDIs: Traumatic dental injuries
